# Supplementary material for: Intensity and Dynamics of Anti-SARS-CoV-2 Immune Responses after BNT162b2 mRNA Vaccination: Implications for Public Health Vaccination Strategies
Source: Vaccines (Basel). 2022 Feb 17;10(2):316. doi: 10.3390/vaccines10020316 (PMC8879063; doi:10.3390/vaccines10020316)
Supplement: Supplementary file 1 [file vaccines-10-00316-s001.zip › vaccines-1574781-supplementary.pdf]

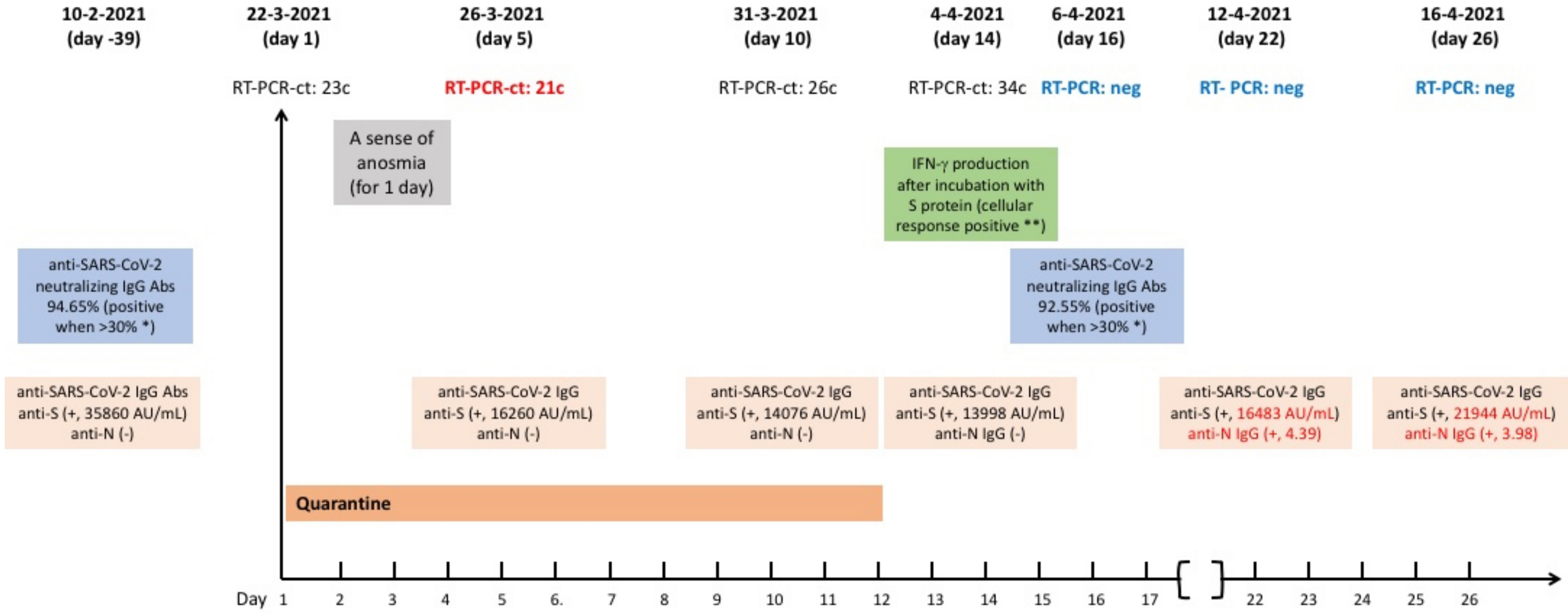

Figure S1: Clinical and laboratory findings of COVID-19 in a 56-year-old female (No #101, VI101) infected post-vaccination. She displayed a medical history of rheumatoid arthritis the last 13 years, diabetes mellitus type 2, hypothyroidism and obesity (body mass index: 38.92). After confirmed COVID-19 infection in her son and 2 months after completion of her vaccination schedule, she underwent an RT-PCR for SARS-CoV-2 via pharyngeal swab and she was found positive. The laboratory analyses performed are presented in detail in Figure, while she com-plained only about a sense of anosmia for one day. She exhibited RT-PCR negativity on day 16 after diagnosis, while on day 22 she displayed for the first-time anti-N anti-SARS-CoV-2 IgG an-tibodies, along with an increase of anti-S anti-SARS-CoV-2 IgG antibodies in her serum; this pat-tern remained similar thereafter, that was indicative of an immune response against the virus. Additional laboratory studies were also performed, revealing the presence of neutralizing an-ti-SARS-CoV-2 antibodies (using the "cPassTM SARS-CoV-2 Neutralization Antibody Detection Kit", Genscript, Leiden, the Netherlands [*]), the detection of adequate anti-SARS-CoV-2 cellular responses (detected as described in Methods [**]) and the B.1.1.7 lineage of SARS-CoV-2 (namely the United Kingdom/Kent variant)

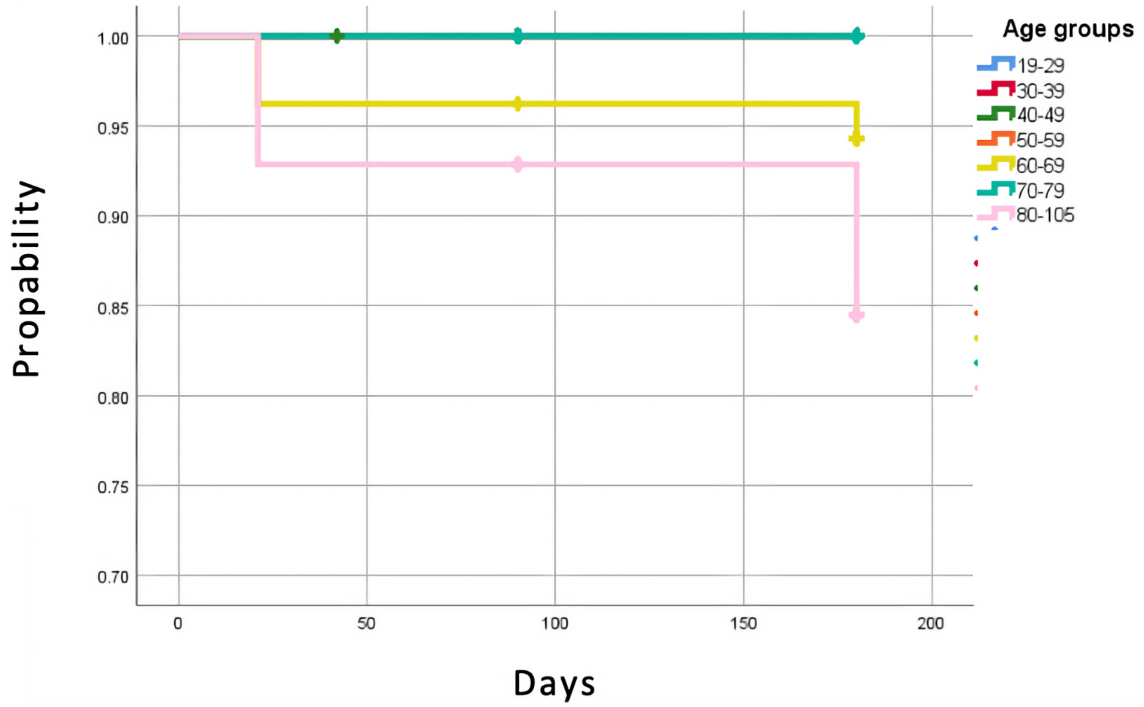

Figure S2: Kaplan-Meier curve of IgG anti-SARS-CoV-2 antibody loss after vaccination according to the age group of enrolled participants

Table S1. Intensity of anti-SARS-CoV-2 IgG responses on day 21 after the first dose of BNT162b2 vaccination

Parameter	N, median IgG levels (mg/dL) (IQR)	*p*, univariate analysis	*p*, multivariate analysis	Coefficient, 95% CI
Sex	Male: 178, 397.1 (1448.0)<br>Female: 286, 444.7 (840.6)	0.525	-	
Age (years)	ρ = -0.453	**<0.001**	**<0.001**	-19.60,<br>-26.36 - -12.85
Hypertension	Yes: 134, 190.9 (640.6)<br>No: 194, 536.3 (1991.5)	**<0.001**	0.570	71.59,<br>-176.09 - 319.28
Diabetes mellitus	Yes: 54, 209.1 (550.1)<br>No: 410, 459.7, (951.1)	**0.001**	0.832	-32.36,<br>-332.29 - 267.57
Dyslipidemia	Yes: 83, 201.9 (477.5)<br>No: 381, 499.3 (1030.5)	**<0.001**	0.577	-69.4,<br>-313.72 - 174.92
Chronic heart disease	Yes: 60, 193.1, (742.8)<br>No: 404, 469.3 (934.3)	**0.003**	0.449	-113.24,<br>-407.06 - 180.59
Stroke/TIA	Yes: 22, 178.0 (527.8)<br>No: 442, 440.4 (942.3)	**0.014**	0.432	-171.84,<br>-601.05 - 257.37
Venous/arterial thrombosis	Yes: 10, 386.5 (1070.4)<br>No: 454, 426.4 (936.6)	0.843	-	
Chronic respiratory disease	Yes: 24, 208.8 (1050.7)<br>No: 440, 439.4 (932.6)	0.167	0.667	87.74,<br>-312.53 - 488.02
Autoimmune/ autoinflammatory diseases	Yes: 38, 341.4 (669.2)<br>No: 426, 439.4 (966.9)	0.655	-	
Thyroid disease	Yes: 56, 504.7 (836.0)<br>No: 408, 415.8 (953.5)	0.450	-	
Chronic liver disease	Yes: 5, 106.1 (344.0)<br>No: 459, 433.8 (935.8)	0.064	0.471	-318.75,<br>-1188.2 - 550.7
Chronic kidney diseases	Yes: 6, 76.2 (7339.9)<br>No: 458, 434.8 (934.0)	0.123	0.859	-80.80,<br>-973.21 - 811.6
Cancer	Yes: 21, 271.5 (1133.1)<br>No: 443, 438.9 (930.6)	0.420	-	
Insomnia/ psychiatric disorders	Yes: 101, 198.7 (1153.0)<br>No: 363, 470.3 (886.6)	**0.007**	0.966	-5.51,<br>-257.4 - 246.39
Other	Yes: 135, 173.7 (635.0)<br>No: 329, 532.4 (1004.2)	**<0.001**	0.567	69.84,<br>-170.03 - 309.72
COVID-19 history	Yes: 50, 12800.5 (30393.1)<br>No: 414, 367.0 (718.1)	**<0.001**	Patients with a history of COVID 19 were not included in multivariate analysis	

Abbreviations: IQR, interquartile range. 95%CI, 95% confidence interval
An explanation of the nature of the clinical parameters (namely, the medical history of the enrolled participants) is presented in detail in Table 1. A multivariate analysis was performed for parameters with a significance p < 0.4 in univariate analysis

Table S2. Intensity of anti-SARS-CoV-2 IgG responses on day 42 after the first dose of BNT162b2 vaccination

Parameter	N, median IgG levels (mg/dL) (IQR)	*p*, univariate analysis	*p*, multivariate analysis	Coefficient, 95% CI
Sex (male)	Male:181, 8902 (16190)<br>Female:294, 9462, (11759)	0.789	-	
Age (years)	ρ = -0.453	**<0.001**	**<0.001**	-170.13,<br>-232.77 - -107.48
Hypertension	Yes: 136, 4805.2, (9747.1)<br>No: 339, 10654.7 (12571)	**<0.001**	0.762	-354.12,<br>-2646.49 - 1938.25
Diabetes mellitus	Yes: 56, 5573.7 (8384.3)<br>No: 419, 9848.8 (13204.8)	**0.003**	0.653	-645.63,<br>-3466.56 - 2175.31
Dyslipidemia	Yes: 84, 5304.0 (8891.1)<br>No: 391, 10188.9 (13223.7)	**<0.001**	0.270	-1274.16,<br>-3540.4 - 992.07
Chronic heart disease	Yes: 61, 5575.5 (12637.1)<br>No: 414, 9463.0 (13161.9)	**0.038**	0.300	-1453.18,<br>-4208.84 - 1302.48
Stroke/TIA	Yes: 25, 4577.7 (8220.3)<br>No: 450, 9369.5 (13280.0)	**0.015**	0.413	-1605.24,<br>-5456.78 - 2246.31
Venous/arterial thrombosis	Yes: 11, 10575.8 (33957.9)<br>No: 464, 9154.85 (13003.7)	0.993	-	
Chronic respiratory disease	Yes: 25, 4141.6 (19620.0)<br>No: 450, 9283.25 (12487.9)	0.383	0.083	3271.63,<br>-427.97 - 6971.23
Autoimmune/ autoinflammatory diseases	Yes: 38, 6282.8 (8611.5)<br>No: 437, 9354.4 (13618.6)	0.209	0.113	-2367.31,<br>-5294.91 - 560.3
Thyroid disease	Yes: 57, 7843.5, (9832.8)<br>No: 418, 9342.95 (39999.8)	0.856	-	
Chronic liver diseases	Yes: 5, 7209.8 (10583.8)<br>No: 470, 9283.25 (13083.9)	0.479	-	
Chronic kidney diseases	Yes: 6, 6595.3 (21678.2)<br>No: 469, 9270.9 (12916.6)	0.717	-	
Cancer	Yes: 23, 9331.5 (32366.7)<br>No: 452, 9154.9 (12583.7)	0.939	-	
Insomnia/ psychiatric disorders	Yes: 103, 5679.3 (12700.1)<br>No: 372, 9941.7 (12790.5)	**0.005**	0.578	-659.49,<br>-2991.03 - 1672.05
Other	Yes: 135, 5258.7 (12114.1)<br>No: 340, 10442.3 (12340.0)	**<0.001**	0.832	-240.44,<br>-2464.5 - 1983.61
COVID-19 history	Yes: 59, 22136.0 (30151.2)<br>No: 416, 7805.4 (11033.7)	**<0.001**	Patients with a history of COVID 19 were not included in multivariate analysis	

Abbreviations: IQR, interquartile range; 95%CI, 95% confidence interval
An explanation of the nature of the clinical parameters (namely, the medical history of the enrolled participants) is presented in detail in Table 1. A multivariate analysis was performed for parameters with a significance p < 0.4 in univariate analysis

Table S3. Intensity of anti-SARS-CoV-2 IgG responses on day 90 after the first dose of BNT162b2 vaccination

Parameter	N, median IgG levels (mg/dL) (IQR)	*p*, univariate analysis	*p*, multivariate analysis	Coefficient, 95% CI
Sex (male)	Male:155, 2097.2 (4599.9)<br>Female: 252, 2198.4 (3092.4)	0.937	-	
Age (years)	ρ = -0.296	**<0.001**	**<0.001**	-39.44,<br>-59.37 - -19.51
Hypertension	Yes: 124, 1543.1 (2944.5)<br>No: 283, 2483.2 (3548.1)	**<0.001**	0.857	67.2,<br>-666.48 - 800.87
Diabetes mellitus	Yes: 60, 1183.2 (2499.0)<br>No: 347, 2271.7 (3467.8)	**0.003**	0.795	-114.99,<br>-984.01 - 754.02
Dyslipidemia	Yes: 82, 1275.1 (2628.1)<br>No: 325, 2291.9 (3770.9)	**<0.001**	0.331	-349.23,<br>-1054.53 - 356.07
Chronic heart disease	Yes :62, 1601.5 (5020.8)<br>No: 345, 2181.9 (3285.1)	0.152	0.783	-125.71,<br>-1023.07 - 771.66
Stroke/TIA	Yes: 23, 1295.3 (1835.4)<br>No: 384, 2181.0 (3592.1)	**0.016**	0.349	-566.14,<br>-1754.96 - 622.69
Venous/arterial thrombosis	Yes: 7, 947.6 (3749.3)<br>No: 400, 2157.9 (3485.8)	0.162	0.210	838.38,<br>-474.77 - 2151.53
Chronic respiratory disease	Yes: 22, 2167.0 (6547.2)<br>No: 385, 2148.8 (3325.0)	0.956		
Autoimmune/ autoinflammatory diseases	Yes: 33, 1705.9 (1694.0)<br>No:374, 2201.7 (3622.5)	0.150	**0.029**	-1076.75,<br>-2044.3 - -109.19
Thyroid disease	Yes: 44, 2220.6 (3061.1)<br>No: 363, 2143.6 (3574.2)	0.689		
Chronic liver diseases	Yes: 4, 1335.3 (1241.0)<br>No:403, 2157.8 (3519.3)	0.324	0.601	-707.43,<br>-3366 - 1951.15
Chronic kidney diseases	Yes: 7, 2254.5 (15541.3)<br>No: 400, 2148.8 (3419.2)	0.874	-	
Cancer	Yes: 18, 1838.7 (3386.1)<br>No: 389, 2157.8 (3477.3)	0.651	-	
Insomnia/ psychiatric disorders	Yes: 126, 1932.4 (5623.2)<br>No: 281, 2191.4 (2988.4)	0.290	0.291	-370.41,<br>-1059.56 - 318.74
Other	Yes: 140, 1533.7 (3511.0)<br>No: 267, 2345.4 (3488.2)	**0.001**	0.861	59.96,<br>-612.9 - 732.82
COVID-19 history	Yes: 54, 9390.4 (26600.6)<br>No: 353, 1911.7 (2875.7)	**<0.001**	Patients with a history of COVID 19 were not included in multivariate analysis	

Abbreviations: IQR, interquartile range; 95%CI, 95% confidence interval
An explanation of the nature of the clinical parameters (namely, the medical history of the enrolled participants) is presented in detail in Table 1. A multivariate analysis was performed for parameters with a significance $p < 0.4$ in univariate analysis

Table S4. Intensity of anti-SARS-CoV-2 IgG responses on day 90 after the first dose of BNT162b2 vaccination

Parameter	N, median IgG levels (mg/dL) (IQR)	*p*, univariate analysis	*p*, multivariate analysis	Coefficient, 95% CI
Sex (male)	Male: 106, 536.8 (1231.9)<br>Female: 216, 727.6, (1011.9)	0.179	0.466	115.37,<br>-195.89 - 426.63
Age (years)	ρ = -0.009	**<0.001**	**0.004**	-16.04,<br>-26.91 - -5.17
Hypertension	Yes: 77, 303.3 (606.3)<br>No: 245, 796.0 (1232.3)	**<0.001**	0.987	3.52,<br>-411.24 - 418.28
Diabetes mellitus	Yes: 41, 308.8 (711.4)<br>No: 281, 745.9 (1141.1)	**0.002**	0.624	-132.57,<br>-665.31 - 400.16
Dyslipidemia	Yes: 61, 329.7 (675.8)<br>No: 261, 771.1 (1217.9)	**<0.001**	0.606	-106.25,<br>-511.25 - 298.74
Chronic heart disease	Yes: 43, 485.5 (956.8)<br>No: 279, 686.7 (1135.5)	0.063	0.687	-101.82,<br>-599.23 - 395.58
Stroke/TIA	Yes: 16, 274.7 (361.1)<br>No: 306, 713.0 (1102.9)	**0.006**	0.854	-68.38,<br>-801.23 - 664.46
Venous/arterial thrombosis	Yes: 3, 796.0 (-)<br>No: 319, 666.0 (1093.8)	0.903	-	
Chronic respiratory disease	Yes: 18, 1077.7 (1627.0)<br>No: 304, 655.2 (1074.6)	0.924	-	
Autoimmune/ autoinflammatory diseases	Yes: 21, 472.8 (951.3)<br>No: 301, 686.7 (1094.4)	0.299	0.538	175.3,<br>-384.43 - 735.03
Thyroid disease	Yes: 35, 711.3 (805.6)<br>No: 287, 660.8 (1110.2)	0.423	-	
Chronic liver diseases	Yes: 1<br>No: 321, 669.2 (1094.4)	0.404	-	
Chronic kidney diseases	Yes: 5, 1155.1 (9168.3)<br>No: 317, 666.0 (1091.4)	0.419	-	
Cancer	Yes: 13, 1448.6 (15083.8)<br>No: 309, 651.3 (1013.6)	**0.035**	0.965	-26.9,<br>-1232.72 - 1178.92
Insomnia/ psychiatric disorders	Yes: 80, 391.8 (927.4)<br>No: 242, 769.6 (1112.8)	**<0.001**	0.849	-39.44,<br>-446.38 - 367.49
Other	Yes: 94, 290.8 (783.9)<br>No: 228, 793.6 (1166.9)	**<0.001**	0.878	-30.55,<br>-420.79 - 359.69
COVID-19 history	Yes: 35, 4056.5 (15302.3)<br>No: 287, 598.8 (880.9)	**<0.001**	Patients with a history of COVID 19 were not included in multivariate analysis	

Abbreviations: IQR, interquartile range; 95%CI, 95% confidence interval

An explanation of the nature of the clinical parameters (namely, the medical history of the enrolled participants) is presented in detail in Table 1. A multivariate analysis was performed for parameters with a significance p < 0.4 in univariate analysis

Table S5. Adverse side effects and intensity of IgG antibody responses on day 21

Parameter	N, median IgG levels (mg/dL) (IQR)	*p*, univariate analysis	*p*, multivariate analysis	Coefficient, 95% CI
Sex (male/female)	Male:178, 397.1 (1448.0)<br>Female: 286, 444.7 (840.6)	0.525		
Age	ρ =-0.453	**< 0.001**	**0.003**	-20.05,<br>-33.25 - -6.86
Comorbidities<br>(≤2 vs ≥3)	>= 3:78, 157.6, (578.8)<br>No: 196, 670.8 (1074.0)	**< 0.001**	0.863	18.36,<br>-191.43 - 228.15
Local pain	Yes: 134, 590.4 (973.1)<br>No: 228, 415.3 (888.5)	**0.005**	0.833	33.25,<br>-277.91 - 344.4
Fever	Yes: 11, 609.3 (2472.8)<br>No: 453, 416.5 (940.0)	0.144	0.563	-255.08,<br>-1122.34 - 612.17
Myalgias	Yes: 11, 1248.7 (1217.0)<br>No: 351, 467.9 (879.4)	**0.038**	0.193	556.6,<br>-284.17 - 1397.37
Fatigue	Yes: 19, 662.1 (918.1)<br>No: 343, 468.5 (901.2)	0.175	0.607	-177.72,<br>-857.36 - 501.92
Headache	Yes: 29, 470.3 (747.3)<br>No: 333, 477.7 (920.8)	0.979	-	
Flu-like symptoms	Yes: 1<br>No: 361, 470.3 (900.8)	0.746	-	
Others	Yes: 24, 687.3 (1252.0)<br>No: 338, 468.2 (887.9)	0.447	-	

Abbreviations: IQR, interquartile range. 95%CI, 95% confidence interval. The comorbidities as presented in detail in Table 1, while the side effects are those emerged after the first dose.

* Others (adverse side effects) are described in detail in Table 2

Table S6. Intensity of anti-SARS-CoV-2 IgA responses on day 21 after the first dose of BNT162b2 vaccination

Parameter	N, median IgG levels (mg/dL) (IQR)	*p*, univariate analysis	*p*, multivariate analysis	Coefficient, 95% CI
Sex	Male: 153 12.1 (23.0)<br>Female: 241, 7.2 (11.0)	**<0.001**	**0.019**	-2.53,<br>-4.65 - -0.42
Age (years)	ρ = -0.280	**<0.001**	**0.001**	-0.131,<br>-0.207 - -0.056
Hypertension	Yes: 112, 6.4, (17.7)<br>No: 282, 9.6, (14.0)	**0.018**	0.727	-0.13,<br>-0.21 - -0.06
Diabetes mellitus	Yes: 43, 4.6 (12.2)<br>No: 351, 9.5, (15.3)	**0.011**	0.139	0.50,<br>-2.33 - 3.34
Dyslipidemia	Yes: 80, 5.3 (10.2)<br>No: 314, 9.9 (15.6)	**0.004**	0.977	-2.64,<br>-6.14 - 0.86
Chronic heart disease	Yes: 50, 5.8 (22.0)<br>No: 344, 8.8 (14.7)	0.265	0.940	-0.04,<br>-2.64 - 2.57
Stroke/TIA	Yes: 21, 7.8 (18.8)<br>No: 373, 8.7 (15.2)	0.644	0.974	0.13,<br>-3.29 - 3.55
Venous/arterial thrombosis	Yes: 6, 1.5 (35.2)<br>No: 388, 8.7, (15.3)	0.386	0.385	-2.15,<br>-7.01 - 2.71
Chronic respiratory disease	Yes: 18, 7.9 (13.3)<br>No: 376, 8.6 (15.4)	0.559	-	
Autoimmune/ autoinflammatory diseases	Yes: 27, 6.6 (9.3)<br>No: 367, 9.0 (15.4)	0.064	**0.028**	-4.32,<br>-8.16 - -0.47
Thyroid disease	Yes: 45, 6.2 (16.6)<br>No: 349, 8.7 (15.3)	0.723		
Chronic liver disease	Yes: 3, 19.5 (-)<br>No: 391, 8.6 (15.3)	0.443	-	
Chronic kidney diseases	Yes: 5, 19.3 (19.7)<br>No: 389, 8.5 (15.1)	0.296	0.208	6.26,<br>-3.49 - 16.02
Cancer	Yes: 13, 7.1 (17.7)<br>No: 381, 8.6 (15.3)	0.820	-	
Insomnia/ psychiatric disorders	Yes: 99, 6.86 (25.5)<br>No: 295, 8.8, (13.2)	0.532	-	
Other	Yes: 127, 6.2 (18.5)<br>No: 267, 10.0 (13.9)	**0.018**	0.839	0.27,<br>-2.37 - 2.92
COVID-19 history	Yes: 43, 36.6 (13.4)<br>No: 351, 7.8 (12.3)	**<0.001**	Patients with a history of COVID 19 were not included in multivariate analysis	

Abbreviations: IQR, interquartile range. 95%CI, 95% confidence interval
An explanation of the nature of the clinical parameters (namely, the medical history of the enrolled participants) is presented in detail in Table 1. A multivariate analysis was performed for parameters with a significance p < 0.4 in univariate analysis
